# Characteristics of Rest and Postural Tremors in Parkinson’s Disease: An Analysis of Motor Unit Firing Synchrony and Patterns

**DOI:** 10.3389/fnhum.2018.00179

**Published:** 2018-05-01

**Authors:** Orsalia M. Agapaki, Constantinos N. Christakos, Dimitrios Anastasopoulos

**Affiliations:** ^1^Laboratory of Systems Physiology, Division of Basic Sciences, Medical School, University of Crete, Heraklion, Greece; ^2^Computational Neuroscience Group, Institute of Applied and Computational Mathematics, Foundation for Research and Technology – Hellas, Heraklion, Greece; ^3^Laboratory of Physiology and Clinical Neurophysiology, School of Nursing, National and Kapodistrian University of Athens, Athens, Greece; ^4^Department of Neurology, Medical School, University of Ioannina, Ioannina, Greece

**Keywords:** Parkinsonian tremor, motor unit spike doublets/triplets, spectral analysis, correlation analysis, synchrony, spinal stretch reflex, beta oscillations

## Abstract

The neural mechanisms responsible for resting and postural tremor in Parkinson’s disease (PD) have been the object of considerable study, much of it focusing on supraspinal sites. Here, we adopted an alternative approach that emphasizes motor unit (MU) firing synchrony and patterns of discharge. To explore if these could account for known features of PD tremor, we recorded the instantaneous acceleration of the upper limb of 23 PD patients at rest or while they tried to hold a stable posture together with surface EMG and single MU discharges of upper limb muscles. Spectral, coherence and cross-correlation analyses of the recorded signals demonstrated alternating epoch-I and epoch-II intervals in PD patients both at rest and while they held a stable posture. Epoch-II intervals are characterized by the presence of 4–8 Hz overt tremor, enhanced MU synchrony and spike-doublets or triplets bearing a one-to-one relation to each tremor cycle. Epoch-I resembled physiological tremor in that it was characterized by 6–10 Hz non-overt tremor, lower MU synchrony and rhythmical MU firing at the intrinsic rate of the unit. The frequency of overt and non-overt tremor remained the same whether the patient was at rest or held a stable posture and the same was true of the remaining characteristics of epoch-I and epoch-II. The mean interval between spikes of a doublet/triplet varied between 30 and 50 ms and, for any given patient, remained roughly constant throughout measurements. This is the first time that enhanced MU synchrony and spike doublets/triplets characterized by relatively stable interspike intervals, are shown to accompany the overt tremor of PD patients. To account for our findings we propose that a two-state oscillatory spinal stretch reflex loop generates overt parkinsonian tremor in response to intermittent, descending, relatively high frequency oscillatory signals.

## Introduction

Rest tremor is one of the cardinal signs of PD. Postural tremor is often present as well, both occurring in the 4–8-Hz range (Bain, 2002). Rest tremor, evaluated while patients are asked to relax, has been studied for the most part separately from postural tremor. The latter coexists with muscle activity opposing gravity. Several investigators have tried to identify the neural mechanisms underlying various forms of limb tremors in PD (Louis et al., 2001; Helmich et al., 2012). Most investigations of Parkinsonian tremor focused on supra-spinal sites (i.e., thalamus, basal ganglia, motor cortex; Lenz et al., 1988; Brown, 2003; Moran et al., 2008; Hirschmann et al., 2013) and assumed that rhythmical cell activation in these regions at frequencies between 4 and 8 Hz are involved in tremor generation. In a few cases, this view was corroborated by coherence observations between such cerebral rhythms and tremor (Zirh et al., 1998; Hurtado et al., 1999; Timmermann et al., 2003). Other investigations, however, obtained results strongly suggesting the involvement of proprioceptive input and peripheral loops or spinal motor systems in tremor generation (Rack and Ross, 1986; Burne, 1987; Spiegel et al., 2002; Rivlin-Etzion et al., 2008). Although hypotheses assuming their supra-spinal origin are far more popular, the neural basis of PD tremor remains unclear.

Whether PD tremor can be due to spinal or supra-spinal of mechanisms, it and other limb tremors could be generated, or at least modified, because of the inertial and viscoelastic properties of moving body segments. For example, it has been argued that physiological finger and hand tremors are almost entirely due to resonance effects (Lakie et al., 2012; Vernooij et al., 2013). Consistent with this, load-dependent frequencies have been reported for the physiological tremor of both the finger and the hand (Stiles and Randall, 1967; Stiles, 1980). Interestingly, several studies observed twin peaks on the power spectrum of PD hand tremor (Stiles and Pozos, 1976; Walsh, 1979; Homberg et al., 1987; Burne et al., 2004) but only one of the two, presumably the mechanical one, is load dependent (Stiles and Randall, 1967; Stiles, 1980).

In order to gain further insight into tremor generating mechanisms in PD, we examined the rhythmical firing synchrony and patterns of MUs in upper-limb muscles during force, rest and postural tremors. Two important reasons guided our approach: first, MU firing synchrony and patterns underlie the formation of force tremors and their features and, hence, the neurogenic components of limb tremors (Christakos et al., 2006). Second, as the MU firing synchrony and patterns ultimately reflect converging neural influences, they may carry information about the tremor-related synaptic input to MNs. This information may be important for exploring the PD tremor generator. Previous work from this group (Christakos et al., 2009) demonstrated that during quasi-isometric muscle contractions in PD patients exhibiting intermittently, overt 4–8 Hz force tremor: (i) the tremor-related synchrony of MUs is enhanced; and (ii) spike-doublets and triplets, with fairly fixed mean interspike interval (ISI), are locked to the tremor. The present study focuses on the detection and exploration of such characteristics of MU synchrony and discharge patterns during rest and postural tremors. Moreover, it examines whether these two tremor types, which occur under quite different conditions, differ in terms of the enhancement of MU synchrony and the prevalence of spike-doublets and triplets.

## Materials and Methods

### Patients and Control Subjects

Twenty-three patients (aged between 52 and 82 years, mean 69 years; male:female, 14:9) with idiopathic PD of tremor-dominant type participated in the study (**Table 1**). They were mild to moderately affected (UPDRS scores in the ‘off’ state, before measurements 7–48, mean 19; Hoehn and Yahr stages I, II, or III). They had been diagnosed between 6 and 96 months (mean 38) before testing. Eighteen age-matched (male:female, 12:6), normal volunteers were the control subjects. All participants gave informed consent. The study conformed to the standards of the latest version of the Declaration of Helsinki, and was approved by the Ethical Committee of the University of Athens.

**Table 1:** Profile of patients with Parkinson’s disease.

Patient	Disease duration since diagnosis (months)	Most affected upper limb	UPDRS			Hoehn and Yahr score	Anti-parkinsonian medication
			Motor score part III	Rest hand tremor item 20	Postural limb tremor item 21		
1	90	Left	42	3	1	3	Levodopa (100+25) mg, 2 tabs
2	78	Left	32	3	3	2.5	Levodopa (100+25) mg, 2 tabsRotigotine 4 mg, 1 patch
3	84	Right	32	3	2	3	Levodopa (100+25) mg, 2 tabs
4	60	Right	36	2	3	2.5	Levodopa (100+25) mg, 2 tabsRotigotine 4 mg, 1 patch
5	46	Right	21	2	2	1.5	Biperiden 2 mg, 2 tabsLevodopa (100+25) mg, 2 tabs
6	6	Left	13	2	1	2.5	Levodopa (100+25) mg, 1 tabRasagiline 1 mg, 1 tab
7	12	Left	9	1	1	1	No
8	12	Right	8	2	1	2	No
9	12	Left	9	1	1	2	No
10	78	Right	48	2	3	1	Levodopa (100+25) mg, 1 tabCarbidopa/Levodopa/Entacapone (100/25/200) mg, 4 tabs
11	24	Left	9	1	1	1	Levodopa (100+25) mg, 0.5 tabRopinirole 0.25 mg, 2 tab
12	12	Right	7	2	1	1	No
13	96	Left	17	1	2	1	Carbidopa/Levodopa/Entacapone (100/25/200) mg, 3 tabsRasagiline 1 mg, 1 tab
14	24	Left	10	2	1	1	Rasagiline 1 mg, 1 tab
15	6	Right	7	2	1	2	No
16	90	Left	16	2	2	1	Carbidopa/Levodopa/Entacapone (100/25/200) mg, 3 tabsRasagiline 1 mg, 1 tab
17	24	Right	7	1	1	1	Levodopa (100+25) mg, 1 tab
18	20	Left	21	1	2	1.5	Rasagiline 1 mg, 1 tab
19	6	Left	21	2	2	1.5	No
20	18	Right	22	2	2	2.5	No
21	32	Right	25	1	1	1.5	Amantadine 100 mg, 2 tabs
22	30	Right	18	1	1	1	Levodopa/Carbidopa (100/250) mg, 2 tabsPramipexole 700 mg, 2 tabs
23	12	Left	10	1	1	1.5	No

### Measurements (Athens Laboratory)

In all patients, the clinical evaluation and the recordings were performed after overnight withdrawal of anti-parkinsonian medication. The upper limb tremor at rest or while maintaining stable posture was mostly asymmetrical. Recordings were obtained from the most severely affected side.

In the recording sessions, the subjects assumed a comfortable sitting position in front of a rigid table. They were instructed to (i) completely relax their most affected upper limb and have the hand hanging over the edge of the table (limb at rest, 32 trials in patients); or (ii) extend the whole limb, trying to maintain a steady horizontal posture against gravity (limb in posture, 31 trials in patients and 34 trials in control subjects). Recording at rest or in posture started as soon tremulous movement of the limb became obvious. Both tremor types were recorded using an accelerometer (K-Beam 5210B, Kistler Instruments) attached to the dorsum of the hand or the digits, depending on the segment that trembled most. Simultaneous sEMG (using Ag-AgCl disk electrodes, Kendall ARBO) and intramuscular EMG (using disposable concentric needle electrodes, 0.37 mm, 26G, Alpine) were obtained in each patient from an extensor (carpi radialis, digitorum communis) or flexor (carpi radialis, digitorum superficialis) muscle of the wrist or digits. Record duration was generally 2 min. To examine if our findings, obtained from patients, who were resting or maintained a stable posture could also be seen when they exerted isometric force, in an additional preliminary study, we asked four randomly selected PD patients to press their extended fingers against a force transducer and recorded isometric force tremor. A detailed description of the procedures employed to collect this data can be found in Christakos et al. (2009) while the methods we used to analyze them are described in detail below. The data were digitized with the help of a 16-bit National Instruments DAQ and the program LabView and stored on hard disk for off-line analysis.

### Data Processing and Analyses (Heraklion Laboratory)

Limb position was sampled at 3 kHz and EMG signals at 5 kHz, a sampling rate which is near the upper bound of those used in the field (e.g., 3 kHz: Johnston et al., 2005; 3–5 kHz: McNulty and Cresswell, 2004; 6.4 kHz: Wessberg and Kakuda, 1999). Manual sorting and custom made subroutines in the MATLAB (MathWorks, Natick, MA, United States) environment were used to discriminate single-MU spikes from the background activity. Spike trains were subsequently represented as sequences of zeroes and ones. Then, limb position and EMG signals were low-pass filtered at 250 Hz, and resampled at 500 Hz to avoid aliasing. This procedure is an ideal filtering that does not affect spectral analyses and coherence estimates (Christakos et al., 1984). The following analyses were carried out using MATLAB.

(1) *In the time-domain*:

(a) Cross-correlograms of pairs of signals, namely limb acceleration, sEMG and MU spike train.
(b) Measurement of the incidence of doublets and triplets of MU spikes as a fraction of all spikes (single spikes, doublets, and triplets) in epoch-II intervals.

(2) *In the frequency-domain*, spectral analyses (Wang et al., 2004; Christakos et al., 2006), performed via the fast Fourier transform on pairs of simultaneous activities, included:

(a) Segmentation of the time-series in 2-s segments, combined with mean removal and windowing (Hanning) for each segment;
(b) Computation of the auto-spectra and the cross-spectrum from each segment (frequency-resolution 0.5 Hz);
(c) Estimation of the auto-spectra and the cross-spectra of pairs of signals (limb acceleration, sEMG, MU spike train) by averaging the estimates from the individual segments.

The coherence spectrum was subsequently estimated as the squared modulus of the cross-spectrum divided by the product of the two auto-spectra. This normative (0–1.0) function quantifies the correlation strength between two signals at each frequency. Its significance threshold was set to 99%, determined from the number of segments analyzed and the desired smooth data tapering for leakage suppression (Rosenberg et al., 1989). It is represented by a horizontal dotted line in the coherence plots.

The amplitude of the tremor at the frequency of the primary component was estimated as the square root of the total power (integral under the power spectrum curve) within the frequency-band of the corresponding auto-spectral deflection, derived from the entire 2-min record. The amplitude of the tremor at the frequency of the secondary component was estimated in a similar manner. As the deflection corresponding to the secondary component was often minute relative to the neighboring large, primary component, the central frequencies of the primary and secondary components were confirmed using auto-spectral and coherence estimates separately from epoch-II and epoch-I intervals, respectively. Transition periods were rejected for this analysis.

(3) *Rhythmical MU firing synchrony*:

The method we employed (Christakos, 1994, 1997) was previously applied to synchronous MU rhythms or modulations (e.g., Christakos et al., 2006, 2009; Erimaki and Christakos, 2008). These unit-to-aggregate analyses have been shown to be experimentally and computationally efficient, as they reveal the behavior of a significant fraction of the MU population. They are easy to use in patients with movement disorders as they avoid the discomfort inherent in the simultaneous recording from multiple MUs. Briefly, coherence computations on samples of MU/sEMG (unit/aggregate) pairs were used for:

(a) Detecting such synchrony, since the occurrence of a non-zero coherence in a sample indicates the presence of a correlated subset within the population, to which the given MU belongs.
(b) Estimating the extent of the synchrony (proportion of correlated MUs in a muscle), as the fraction of non-zero coherences.
(c) Obtaining information on the synchrony strength; importantly, for widespread and in-phase synchrony, the MU/MU coherence approaches the squared value of the MU/sEMG coherence (Christakos, 1997) and can thus be easily estimated.
(d) Further, MU/sEMG cross-correlation computations for the coherent MUs in the sample were used for estimating MU phases, in terms of delays relative to the sEMG (common reference signal). Such information on multiple MU phases cannot be obtained from MU/MU analysis which provides information about phase differences between a single MU pair.

The strength of MU to acceleration coherence did not differ significantly from MU to sEMG coherence (*p* = 0.957, paired *t*-test). In relatively few cases the MU/sEMG coherence at the frequency of the secondary component was a little below the significance threshold (99% confidence). These included 14% of the records while patients maintained a stable posture and 20% of those during rest tremor in patients as well as 21% of the records while control subjects maintained a stable posture. Nevertheless, MU/sEMG coherence was significant with 95% confidence in these cases. The sEMG/acceleration coherence provided additional support for the presence of synchrony at the frequency of the secondary component. It is important to note that this aggregate/aggregate coherence analysis, although simple, can be misleading when it comes to estimates of the extent and strength of synchrony. It saturates easily and in cases of widespread and in-phase synchrony it greatly overestimates the true coherence between MUs (Christakos, 1997).

### Statistical Analyses

Statistical analyses were performed using the SPSSv20 package. The normality of the distributions was examined using the one-sample Kolmogorov–Smirnov test. The paired-samples *t*-test was used to compare the means of two distributions in cases when members of the one could be paired to members of the other (Armitage, 1971). This was done when comparing: (1) MU/sEMG and MU/acceleration coherences estimated for the *same* MU spike train, (2) the frequencies of the primary and secondary components obtained from the *same* record, (3) the mean ISI of doublets in different records from the *same* patient, (4) the mean ISI of doublets from a record and the mean short ISI of all records from the *same* patient. The independent-samples *t*-test was used in all other cases. One-way ANOVA was used to evaluate how the mean short ISI is affected when measured in all relevant records obtained from a single patient and from: (i) doublets, when doublets are interspersed with single spikes, (ii) doublets, when doublets are interspersed with triplets and (iii) triplets, when triplets are interspersed with doublets. Univariate ANOVA was used to determine if the ISI is affected by a number of factors (patient, state of the subject such as resting or holding a stable posture, spike grouping in doublets or triplets, whether doublets are interspersed with triplets or single spikes, etc.). Linear regression was used to examine the relationship between the proportion of doublets/triplets and the ratio of the mean firing rate of the MU (*f*_U_) to overt tremor frequency. The threshold for rejecting the null hypothesis was set at *p <* 0.05 in all statistics tests.

## Results

**FIGURE 1:**
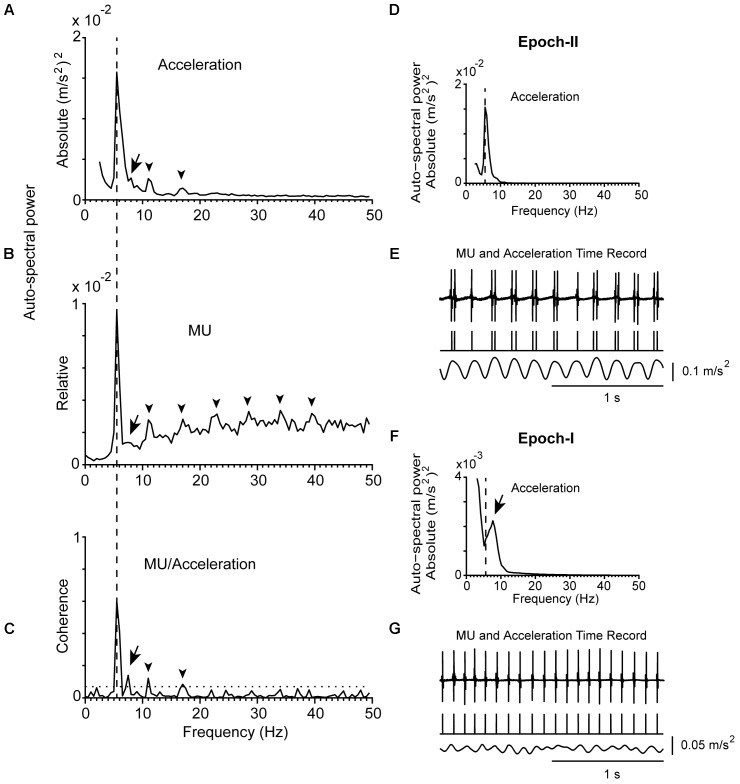
Representative example of the tremor and the firing of a MU observed in a patient (#14) while stable posture was maintained. **(A)** Auto-spectrum of hand acceleration displaying the primary (5.5 Hz, vertical dashed line) and secondary (7.5 Hz, arrow) components of tremor as well as the first two harmonics of the primary component (arrowhead). **(B)** Auto-spectrum of a MU to display the primary (vertical dashed line) and secondary (arrow) components of tremor as well as several harmonics of the primary component (arrowheads). **(C)** MU/Acceleration coherence displaying the primary (vertical dashed line) and secondary (arrow) components of tremor as well as the first two harmonics of the primary component (arrowheads). The horizontal dashed line represents the significance threshold. **(D,F)** Auto-spectra of hand acceleration using only epoch-II **(D)** or epoch-I **(F)** intervals (total duration 67 and 53 s, respectively). Symbols as in **(A)**. Note the absence of the secondary component in **(D)** and of the primary component in **(F)**. **(E,G)** Traces corresponding to the accelerometer raw signal (bottom), the MU spike train (middle) and the intramuscular raw EMG signal (top) recorded simultaneously from the patient during epoch-II **(E)** or epoch-I **(G)** intervals. The mean firing rate of the MU was 10.0 Hz in both **(E,G)**.

**FIGURE 2:**
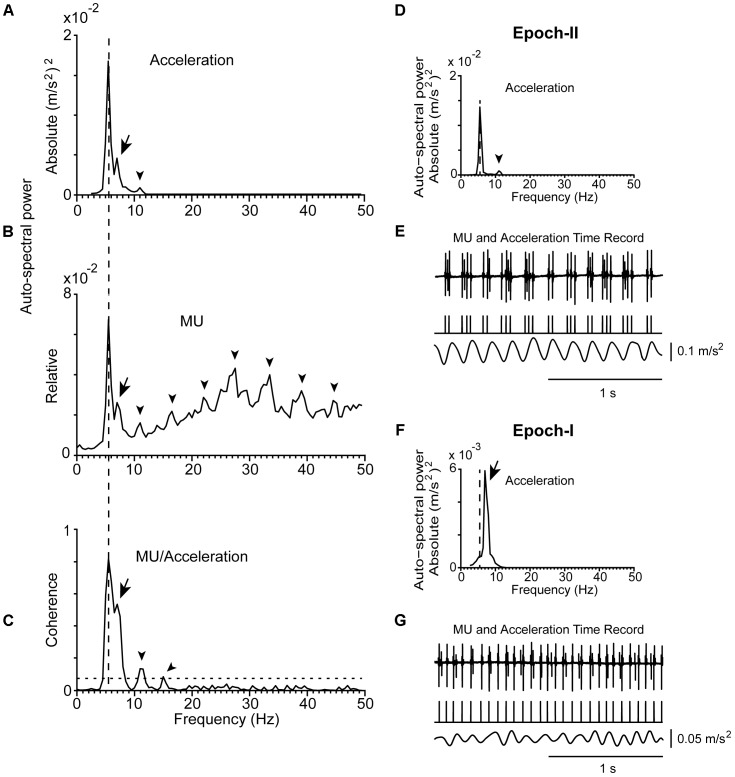
Representative examples of the tremor and the firing of a MU observed in a patient (#21) at rest. **(A)** Auto-spectrum of hand acceleration displaying the primary (5.5 Hz, vertical dashed line) and secondary (7 Hz, arrow) components of tremor as well as the first harmonic of the primary component (arrowhead). **(B)** Auto-spectrum of a MU to display the primary (vertical dashed line) and secondary (arrow) components of tremor as well as several harmonics of the primary component (arrowheads). **(C)** MU/Acceleration coherence displaying the primary (vertical dashed line) and secondary (arrow) components of tremor as well as the first two harmonics of the primary component (arrowheads). **(D,F)** Auto-spectra of hand acceleration using only epoch-II **(D)** or epoch-I **(F)** intervals (total duration 66 and 54 s, respectively). Symbols as in **(A)**. Note the absence of the secondary component in **(D)** and of the primary component in **(F)**. **(E,G)** Traces corresponding to the accelerometer raw signal (bottom), the MU spike train (middle) and the intramuscular raw EMG signal (top) recorded simultaneously from the patient during epoch-II **(E)** or epoch-I **(G)** intervals. The mean firing rate of the MU was 14.0 Hz in both **(E,G)**.

**FIGURE 3:**
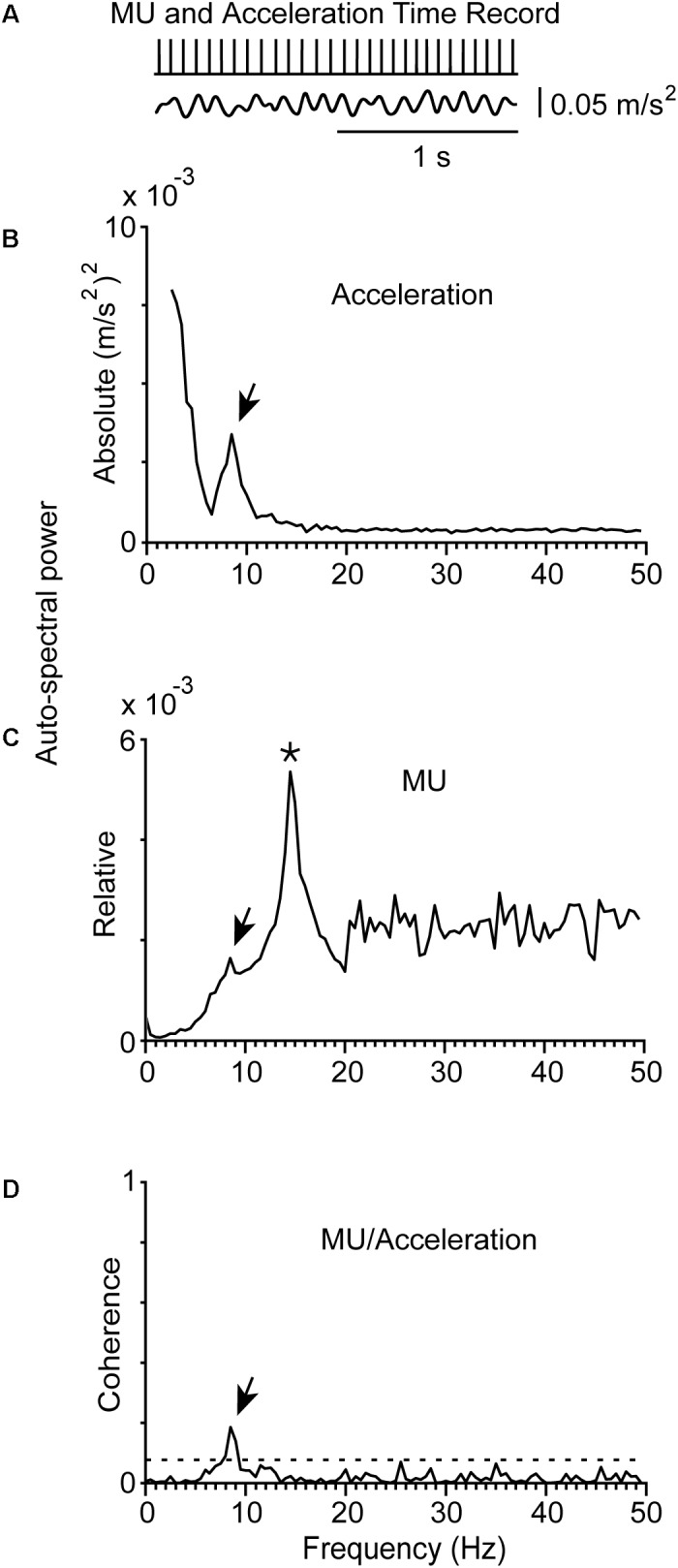
Representative example of physiological tremor and the firing of a MU in a control subject (#5) while maintained stable posture. **(A)** Traces corresponding to the accelerometer raw signal (bottom), the MU spike train (top) recorded simultaneously from the control subject. The rhythmical firing rate of the MU was at 14.5 Hz (mean firing rate of the MU) and the frequency of the tremor at 8.5 Hz. **(B)** Auto-spectrum of the hand acceleration displaying a single peak at the tremor frequency (arrow). **(C)** Auto-spectrum of the MU displaying a large peak at the frequency of the mean firing rate of the MU (asterisk) and a small deflection at tremor frequency (arrow). **(D)** MU/Acceleration coherence displaying a small but significant peak at tremor frequency (arrow).

**FIGURE 4:**
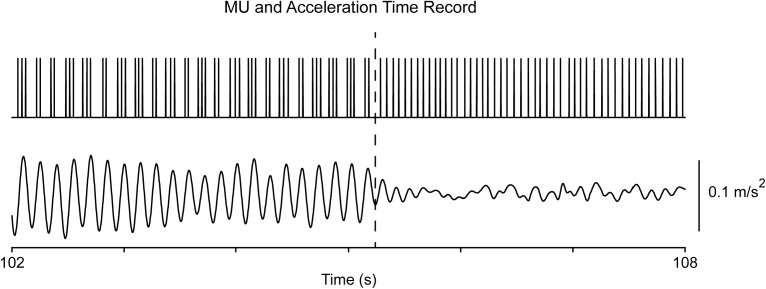
Transition from epoch-II to epoch-I in a patient (#8) at rest. The top trace illustrates the MU spike train and the bottom the acceleration of the hand. Note that the MU firing rate (17 Hz) before the transition (dashes) is identical that after the transition.

As previously reported (Christakos et al., 2009), records obtained from PD patients both at rest and when maintaining a stable posture were characterized by the appearance of overt tremor (epoch-II) randomly interspersed with periods of non-overt tremor (epoch-I). Each epoch could last from around one second to several dozens of seconds. MU firing was normal-like in epoch-I (**Figures 1G**, **2G**), showing a strong, intrinsic rhythm at the *f*_U_ of each MU and, additionally, a weaker rhythm exhibiting low coherence to tremor (**Figure 1C**). There are clear similarities between the characteristics of tremor and the activity of MUs in epoch-I intervals of PD patients (**Figures 1F,G**) on the one hand, and the characteristics of physiological tremor together with the activity of MUs in control subjects (**Figures 3A,B**). In contrast, MU firing in epoch-II was highly abnormal, showing spike-doublets and triplets bearing a one to one relationship to each tremor cycle (**Figures 1E**, **2E**). It is important to note that *f*_U_ remained constant despite epoch transitions (**Figure 4**) which usually lasted less than half a second. Readers can convince themselves that this is true by counting the number of spikes per second before the transition illustrated in **Figure 4** (dashes), which is identical to that after the transition. Similar findings were obtained from force tremor recordings in four patients.

### Components of Tremor

**Figure 1A** provides an example of the power density of the fast Fourier transform of an acceleration record lasting for 2 min. It was obtained from a PD patient while stable posture was maintained and contains both epoch-I and epoch-II intervals. It is characterized by a prominent (primary) component (dashed vertical line) and an additional, harmonically unrelated, smaller peak at a higher frequency (secondary component; **Figure 1A**, arrow). Although the latter was often (*N* = 56) barely discernible (**Figure 1A**, arrow), it was always clearly visible in auto-spectra obtained exclusively from intervals characterized by non-overt tremor (epoch-I; **Figures 1F**, **2F**). In contrast, a large solitary component appeared in tremor auto-spectra obtained exclusively from epoch-II intervals at the relatively low frequency typical of the primary component (compare **Figures 1D** with **1A** and **Figures 2D** with **2A**). Similar results were obtained from another 30 trials during postural and 32 trials during rest tremor obtained from 23 patients.

**FIGURE 5:**
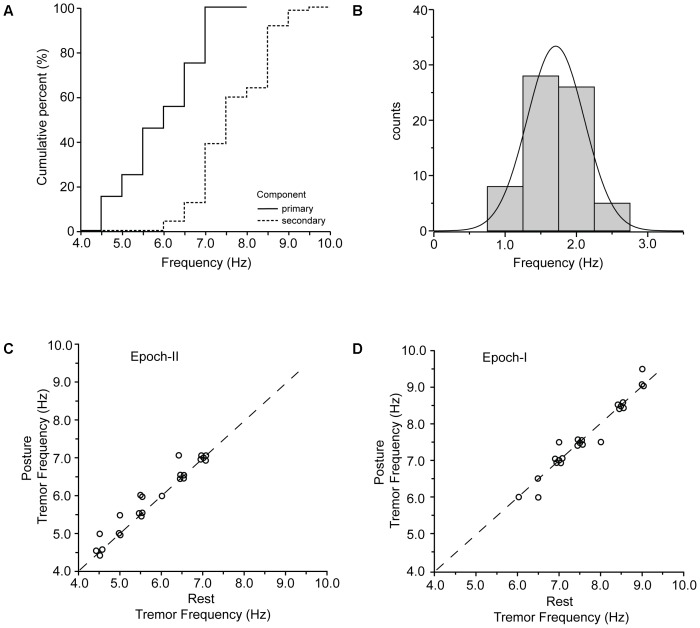
Primary and secondary components of PD tremor. **(A)** Cumulative curves of the frequencies of the primary (solid) and secondary (dashed) components of tremor in the population of PD patients. **(B)** Frequency histogram of the difference between the primary and the secondary component of tremor in all of the records we collected (*N* = 63). **(C,D)** Scatterplot of tremor frequency at rest (abscissa) vs. tremor frequency while holding a stable posture (ordinate) measured from epoch-II **(C)** and epoch-I **(D)** intervals. Each data point is from a different patient.

For both postural and rest tremor, the frequency of the primary component was in the 4–8 Hz range which is typical of the PD tremor. In contrast, the frequency of the secondary component was in the range of the physiological tremor, i.e., between 6 and 10 Hz. The difference between these two ranges is illustrated in the cumulative frequency histogram of **Figure 5A** and is highly significant (*p* < 0.001, paired *t*-test). Taking each patient separately, the secondary component of tremor was on the average 1.72 ± 0.42 Hz (mean ± SD) higher than the primary (**Figure 5B** and **Table 2**). **Figure 5C** plots the relationship between the frequency of the primary component of tremor at rest against the frequency of the primary component of tremor while maintaining a stable posture for each one of the 23 PD patients we studied. As shown here, most of the data (*N* = 18) lies along the diagonal which implies that the frequency of the primary component at rest is identical to that during stable posture. In the remaining five cases the difference did not exceed the resolution of our technique (0.5 Hz). With the exception of four patients, the frequency of the secondary component of tremor at rest was also identical to that during stable posture (**Figure 5D**). The large difference between the primary and secondary components of tremor in terms of power density (**Figures 1A**, **2A**) reflect the large differences between epochs I and II in terms of tremor amplitude. During epoch-I (dominated by the secondary component) the amplitude of the postural (0.164 ± 0.084 m/s^2^, mean ± SD) and rest (0.144 ± 0.112 m/s^2^) tremor in patients was small and comparable to that of the postural tremor in control subjects (0.156 ± 0.072 m/s^2^). In contrast, during epoch-II, dominated by the much larger primary component, the amplitude of the postural and rest tremor of patients was abnormally large (0.958 ± 0.076 and 0.843 ± 0.056 m/s^2^, respectively), exceeding thus the physiological tremor amplitude by a factor of around 6.

**Table 2:** Summary of experimental results.

	**Patients**				**Control subjects**
***f*_U_ (Hz)**					
Range	6.0–18.5				7.0–20.0
Mean (*SD*)	10.9 (2.9)				14.6 (3.4)
Recordings	Rest (*n* = 32)		Postural (*n* = 31)		Postural (*n* = 34)
*n* of MUs	35		31		35
Tremor Component	Primary	Secondary	Primary	Secondary	Single
	
***f*_T_ (Hz)**					
Range	4.5–7.0	6.0–9.0	4.5–7.0	6.0–9.5	6.5–10.0
Mean (*SD*)	5.9 (0.80)	7.7 (0.91)	6.1 (0.84)	7.9 (0.92)	8.2 (0.93)
***A*_T_ (m/s^2^)**					
Range	0.252–2.309	0.015–0.340	0.376–3.085	0.046–0.344	0.034–0.328
Mean (*SD*)	0.843 (0.056)	0.144 (0.112)	0.958 (0.076)	0.164 (0.084)	0.156 (0.072)
**MU/EMGCoh**					
Range	0.23–0.96	0.05–0.75	0.21–0.97	0.04–0.80	0.04–0.77
Mean (*SD*)	0.73 (0.18)	0.20 (0.19)	0.76 (0.16)	0.24 (0.19)	0.19 (0.17)

*A_*T*_, tremor amplitude; f_*U*_, MU mean firing rate; f_*T*_, tremor frequency; MU/EMGCoh, MU/EMG coherence value*.

### Characteristics of the Motor Unit Firing Synchrony

**FIGURE 6:**
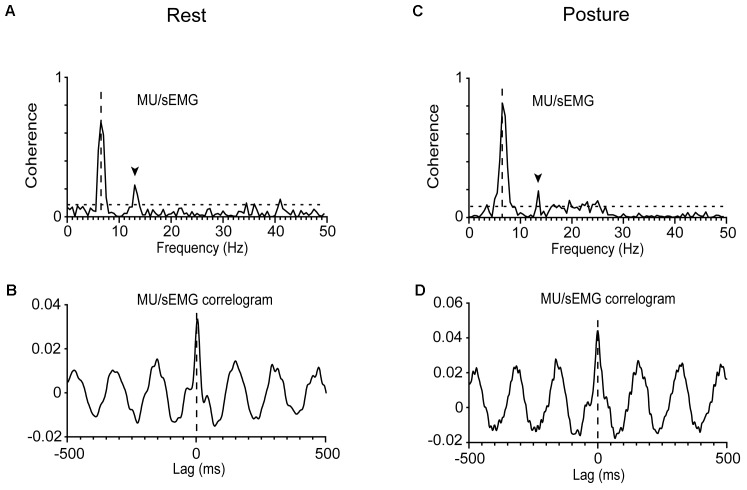
Analysis of MU synchrony in a patient (#12) at rest **(A,B)** or while he maintained a stable posture **(C,D)**. **(A,C)** The very high MU/sEMG coherence at 6.5 Hz. **(B,D)** The zero-lag central peak (in-phase synchrony) in the oscillatory MU/sEMG cross-correlogram at 6.5 Hz.

The strength of the tremor-related MU synchrony, as judged from the coherence of MUs to acceleration and sEMG (see section “Materials and Methods”), varied considerably within the same patient and between patients (**Table 2**). In general, it obtained large values when evaluated at the frequency of the primary component and smaller at the frequency of the secondary. For example, it equals 0.62 when MU to acceleration coherence was evaluated at 5.5 Hz and only 0.13 at 7.5 Hz (**Figure 1C**). Patients displaying increased MU synchrony (i.e., high coherence values at the primary component frequency) during rest also displayed increased MU synchrony while maintaining stable posture. **Figures 6A,C** provide an illustrative example. At the frequency of the primary component (6.5 Hz), the strength of MU to sEMG coherence was 0.70 at rest and 0.81 while maintaining stable posture. In PD patients, the strength of the MU to the sEMG coherence was high at the frequency of the primary component, both at rest and while maintaining stable posture; the former obtained values (0.73 ± 0.18) that did not differ significantly (*p* = 0.543, independent *t*-test) from those of the latter (0.76 ± 0.16). The much smaller values of the MU to sEMG coherence at the frequency of secondary components were also similar (*p* = 0.360, independent *t*-test) at rest (0.20 ± 0.19, **Table 2**) and while maintaining stable posture (0.24 ± 0.19). More importantly, they were similar to those obtained from control subjects maintaining stable posture (0.19 ± 0.17).

The cross-correlation between the spikes of individual MUs and sEMG records indicates that MU discharges were, on average (-2 ± 12 ms, *N* = 66), in phase with the sEMG and thus with one another (see section “Materials and Methods”). **Figures 6B,D** provide typical examples of two different MUs, recorded from the same patient at rest (**Figure 6B**) and while maintaining stable posture (**Figure 6D**), respectively. An estimate of the MU to MU coherence can be obtained by squaring the MU to sEMG coherence (see section “Materials and Methods”). When measured at the frequency of the secondary component, MU to MU coherence equaled 0.10 ± 0.14 in PD patients maintaining a stable posture and 0.08 ± 0.16 in the same patients at rest. Similar values were seen in control subjects 0.08 ± 0.12 maintaining a stable posture. On the contrary, MU synchrony in PD patients was much stronger at the frequency of the primary component as surmised from the MU to MU coherence estimated at rest (0.56 ± 0.23) or while they maintained a stable posture (0.59 ± 0.21).

### Firing Patterns of Motor Units

#### MU Firing Patterns

The mean firing rates of the MUs we studied in PD patients at rest or maintained a stable posture ranged between 6.0 and 18.5 Hz (*f*_U_; **Table 2**). It ranged between 7.0 and 20.0 Hz in control subjects (**Figure 3A**). During epoch-I intervals, each MU displayed rhythmic activity at its intrinsic *f*_U_ (e.g.,**Figures 1G**, **2G**). This rhythm appears as an easily discernible peak in the auto-spectrum of the MU illustrated in **Figure 3C** (asterisk). Additionally, the auto-spectrum of the MU displayed a second component (e.g., **Figures 1B**, **2B**; arrow). On the frequency axis it occupies a position identical to that of the secondary component of the MU to acceleration coherence plot (**Figures 1C**, **2C**; arrow). It also corresponded to the dominant component of the physiological tremor of control subjects maintaining a stable posture (**Figure 3D**).

During epoch-II intervals, and while subjects displayed overt tremor, MUs exhibited abnormal discharge patterns. Twenty of the MUs that we recorded from PD patients while they maintained a stable posture exhibited spike-doublets randomly interspersed with single spikes (e.g., **Figure 1E**). As shown here, either single or double spikes occurred in a one-to-one relation to the tremor cycle. As described before (Christakos et al., 2009), the sum of one short and one long ISI corresponds to the tremor period. In these MUs, *f*_U_ was maintained at a frequency intermediate between that of the tremor and twice its value. Similar data were obtained in 28 MUs recorded while PD patients were at rest. The firing pattern of another 4 (9) MUs recorded from PD patients at rest (while they maintained a stable posture) was a series of spike-triplets randomly interspersed with doublets as shown for example in **Figure 2E**. Once again, either doublets or triplets occurred in a one-to-one relation to tremor cycles. In this case, it is the sum of two short and one long ISI that corresponds to the tremor period (Christakos et al., 2009). The *f*_U_ obtained in these MUs between twice and three times the value of the tremor frequency. In two patients, four MUs exhibited spike-quadruplets interchanged randomly with triplets at rest (two units) or while they maintained a stable posture (the remaining two units). Here, it is the sum of three short and one longer ISI that corresponds to the tremor period. The *f*_U_ of these MUs was higher than three times the value of the tremor frequency. The firing pattern of only one MU recorded at rest exhibited single-spikes in a one-to-one relation to the tremor. Evidently, in this case *f*_U_ equaled the tremor frequency. To summarize, MUs discharged at a rate equal to or higher than that of epoch-II tremor and therefore their recruitment rate is equal to the *f*_T_.

**FIGURE 7:**
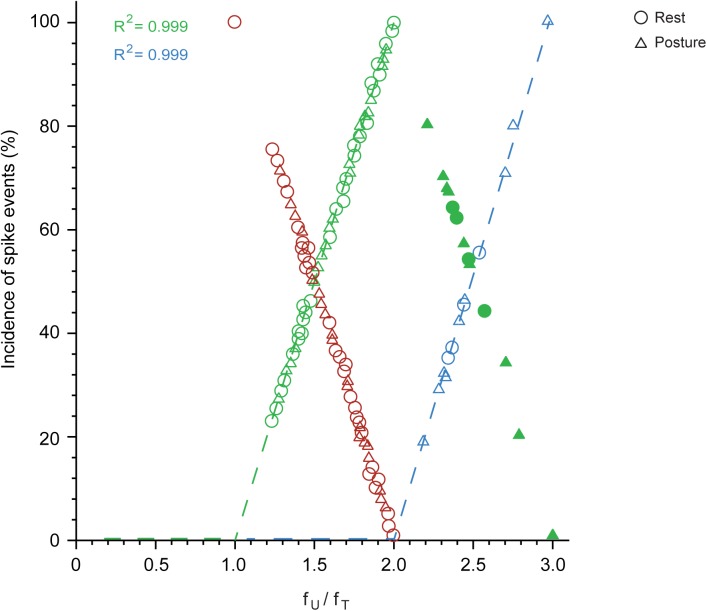
The proportion of single-spikes, spike doublets and triplets, depends on the firing rate of the MU (*f*_U_) and the frequency of the tremor (*f*_T_) during epoch-II intervals. Note the linear relationship between the incidence of single-spikes (red; *N* = 49) and the ratio *f*_U_/*f*_T_. Also note the linear increase of the incidence of doublets (open green; *N* = 48) up to *f*_U_/*f*_T_ = 2 and its decrease above this value (solid green; *N* = 13). Finally note the linear relationship of the incidence of triplets (blue; *N* = 13) to the ratio *f*_U_/*f*_T_.

Spikes are grouped into doublets, triplets, etc. and the proportion of these depends on the firing rate of the unit in relation to the frequency of the tremor. This is shown in **Figure 7**, which plots the proportion of doublets (open green) as a function of the ratio *f*_U_/*f*_T_ (where *f*_T_ is the frequency of the overt tremor). As shown here, the proportion of tremor cycles accompanied by doublets increased linearly together with *f*_U_/*f*_T_ in all 48 of the relevant MUs and equaled 100% (doublets present in all tremor cycles) when *f*_U_ = 2 × *f*_T_. The excellent relationship between the proportion of doublets and the ratio *f*_U_/*f*_T_ is indicated by the fact that it accounts for 99.9% of the variance of the dependent variable. From the regression equation, the proportion of tremor cycles with doublets is (*f*_U_ -*f*_T_)/*f*_T_. Accordingly, *all* spikes in excess of *f*_T_ can be thought of as combining with other spikes to form doublets that are randomly interspersed with the remaining single spikes. As expected, the proportion of tremor cycles accompanied by single-spikes decreased linearly with *f*_U_/*f*_T_ (**Figure 7**, red). Similarly, the proportion of tremor cycles accompanied by triplets (**Figure 7**, blue) increased linearly together with *f*_U_/*f*_T_ in all 13 relevant MUs and again equalled 100% (triplets in all tremor cycles) for *f*_U_ = 3 × *f*_T_. Here again the relationship between the two variables was perfect as it accounted for 99.9% of the variance of the dependent one and once again *all* spikes in excess of 2 × *f*_T_ can be thought of as combining with doublets to form triplets randomly interspersed with the remaining doublets. Here again, the proportion of tremor cycles accompanied by doublets decreased linearly with *f*_U_/*f*_T_ (**Figure 7**, solid green).

#### Interspike Intervals Within Doublets/Triplets

**FIGURE 8:**
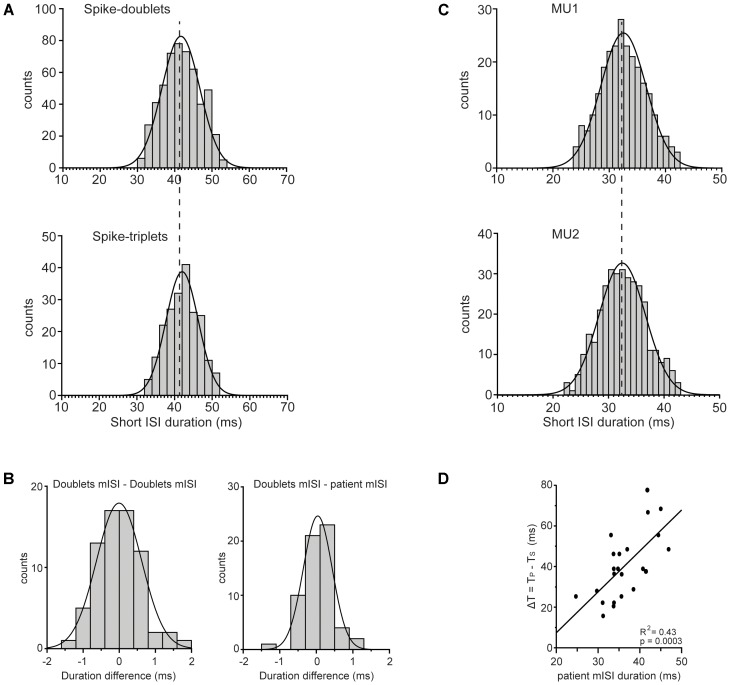
Features of mean ISI. **(A)** Frequency histogram of the duration of the short ISIs measured from the doublets (top; *N* = 526) or the triplets (bottom; *N* = 208) found in the same MU spike train. Note the statistically indistinguishable means (vertical dashed line) of the two distributions (41.6 ± 5.1 ms and 42.0 ± 4.3 ms, respectively). **(B)** Left: Frequency histogram of the difference between the mean ISI (mISI) of doublets found in any 2 min record and that of doublets from any other 2 min record from the same patient. Note that the average difference (–0.005 ± 0.62 ms, *N* = 70) was statistically indistinguishable (*p* = 0.944) from zero. Right: Frequency histogram of the difference between the mISI of doublets from any 2 min record and that of all short ISIs in all records of the same patient. Note that the average difference (–0.038 ± 0.39 ms, *N* = 61) was statistically indistinguishable (*p* = 0.453) from zero. **(C)** Frequency histogram of the short ISIs in doublets (top; *N* = 257) and in doublets/triplets (bottom; *N* = 442) observed in two different MUs that were recorded simultaneously and discharged at a rate of 7.0 Hz (top) and 12.0 Hz (bottom), respectively. Note the statistically indistinguishable means (vertical dashed line) of the two distributions (32.53 ± 4.0 ms and 32.47 ± 4.2 ms, respectively) despite the very different firing rates of the two MUs. **(D)** The difference (Δ*T*, ordinate) of the period of non-overt tremor (*T*_S_) from the period of overt tremor (*T*_P_) depends on the mean short ISI (abscissa) of the patient. Each data point is from a different patient and the line through them is the linear regression line.

The short ISIs within doublets (**Figure 8A**; top) and triplets (**Figure 8A**; bottom) were concentrated around mean values in the 30–50 ms range. The mean ISI of doublets in any 2 min record did not differ significantly from that of the triplets (*p* > 0.10, one-way ANOVA) found in the same record (compare the two plots of **Figure 8A**). Nor did it differ from that of the doublets found in any other 2 min record from the same patient as shown by the fact that the difference between the two (**Figure 8B**; left) was not significantly bigger than zero (*p* = 0.944, paired *t*-test) when we considered all possible pairs of records obtained from all patients. Finally, it did not differ significantly from that of all short ISIs in all records of the same patient again shown by the fact that the difference between the two (**Figure 8B**; right) was not significantly bigger than zero (*p* = 0.453, paired *t*-test). Thus, the mean short ISI in any 2-min record was practically invariant across different records obtained from a patient and thus represented a robust personal characteristic or ‘idiosyncratic’ feature of his tremor-related MU firing. Univariate ANOVA showed statistically significant effect only for the factor ‘patient’ (*F* = 656.064, *P* < 0.0001) and not for other factors such as state of the patient (resting or holding a stable posture), spike grouping (doublets or triplets) and firing pattern (single spikes interspersed with doublets or doublets interspersed with triplets). One might argue that the mean short ISI depends on the excitation level of the unit expressed in its *f*_U_. To examine if this is the case we separated the MUs we encountered into two groups, those with relatively high mean rates (mean ± SD: 13.5 ± 2.3 Hz; range: 11–18.5 Hz, *N* = 28) and those with relatively low mean rates (mean ± SD: 9 ± 1.2 Hz; range: 7–10.5 Hz, *N* = 38) and examined if the mean short ISI of the former is smaller than that of the latter. The average short ISI of MUs characterized by high firing rates was equal to 37.2 ± 5.6 ms while the mean short ISI of MUs characterized by low firing rates was equal to 36.5 ± 4.4 ms. The small difference between the two, albeit in the wrong direction, was insignificant (*p* = 0.58, independent *t*-test). The fact that the mean short ISI does not depend on MU firing rate was also demonstrated in records obtained from three pairs of MUs that were simultaneously recorded in three different patients and which exhibited the same mean short ISI despite large differences in the rates with which they fired at rest. For example, the upper plot of **Figure 8C** illustrates the distribution of the short ISIs seen in a MU that was firing at a rate of 7 Hz while the lower plot, illustrates those seen in a second MU that was firing at a rate of 12 Hz and was recorded at the same time. The mean short ISI of the former (mean ± SD: 32.53 ± 4.0 ms) did not differ significantly (*p* = 0.868, independent *t*-test) from that of the latter (mean ± SD: 32.47 ± 4.2 ms). Interestingly, the ‘idiosyncratic’ mean short ISIs of our 23 patients were nevertheless correlated (*R*^2^: 0.43, *p* = 0.0003) to the difference between the periods that correspond to the primary and secondary components (**Figure 8D**). The implications of this finding are considered in the Discussion together with the possible role of the stretch reflex loop and descending beta-range oscillatory signals in the generation of PD tremor.

## Discussion

Our main results are: (a) The presence of epoch-II intervals randomly interspersed with epoch-I intervals both at rest and while the patients maintained a stable posture. (b) Epoch-II intervals are characterized by enhanced MU synchrony at the frequency of the primary component of tremor and spike doublets/triplets exhibiting fixed, beta-range, patient specific, mean short ISIs bearing a one to one relationship to each tremor cycle. (c) Epoch-I intervals are reminiscent of the physiological tremor of normal subjects and are characterized by rhythmical MU firing and weak MU synchrony at the frequency of the secondary component of tremor. (d) The frequency of the primary and secondary components did not change appreciably whether a patient was resting or maintaining a stable posture.

### Characteristics of PD Tremor and Associated MU Firing

#### Components of PD Tremor

A 6–10 Hz rhythm (defined as the secondary component) appeared only during epoch-I intervals randomly interspersed with epoch-II intervals characterized by the presence of 4–8-Hz overt tremor (defined as the primary component). The secondary component of non-overt tremor, was not a harmonic of the primary PD tremor component. Both at rest and while maintaining a stable posture, it was usually small and often obscured by the primary component. The small amplitude of the secondary component was not due to the small duration of epoch-I since it was roughly the same as that of epoch-II. Moreover, the frequencies of the two components did not differ by more than 1.0–2.5 Hz. This and the small duration of records obtained from patients could shift estimates of the true frequency of parkinsonian tremor from values in the neighborhood of the primary component to values closer to the secondary. As with the primary, the secondary component gave rise to significant unit to aggregate coherences in all of our records. As argued before (Lakie et al., 2012), this implies that the secondary component does not arise from the mechanical resonance of the limb. More generally, interactions of neural tremor generating mechanisms with oscillations due to mechanical resonance seem unlikely because: (i) The frequency of the primary component at rest did not change when patients were asked to maintain a stable limb posture against gravity despite the large differences of the two situations in terms of limb “masses” and the same is true of the secondary component. (ii) The transition from epoch-I to epoch-II intervals and back could not be due to mechanical factors, in particular during isometric force tremor, and therefore is clearly neurogenic.

#### MU Firing

In agreement with previous observations in PD patients exerting isometric force (Christakos et al., 2009), the strength of the widespread, in-phase MU synchrony at the frequency of the primary component of the tremor was higher at rest as well as while patients maintained a stable posture. In contrast, PD patients did not differ from control subjects in terms of MU synchrony at the frequency of the secondary component, largely reflecting MN firing rhythmicity (Christakos, 1982). The occurrence of spike doublets/triplets follows a rule depending on the ratio of *f*_U_/*f*_T_, as also previously described for isometric force tremor in PD patients (Christakos et al., 2009).

#### Significance of ISI Features

Our data demonstrate that the average duration of the short ISIs (i.e., the interval between spikes that belong to a doublet or triplet) is nearly fixed for all tremor types, is idiosyncratic and patient specific, is in the beta range (30–50 ms) and does not depend on the mean firing rate of the unit. It has been argued that such spike-events may represent normal MN firing in response to increased net excitation (Dietz et al., 1974), as for example when strong synaptic input leads to fast contractions (see section “Discussion” in Dengler et al., 1986). If this were the case, the spikes of doublets emitted by smaller MUs, characterized by higher *f*_U_, would be separated by shorter ISIs than those of bigger MUs in response to the same synaptic drive (Granit et al., 1966; Matthews, 1996). As a consequence, the average duration of the short ISIs would drop for MUs with high *f*_U_, and this we did not observe. It would also be shorter for the triplets relative to the doublets emitted by a certain unit, the quadruplets relative to the triplets, etc. Our findings demonstrate that these expectations do not reflect reality either. The coherent 4–8 Hz rhythms found in the brain could instead represent ascending projections of muscle spindles and would thus co-vary with the tremor (Hirschmann et al., 2013). This is consistent with the results of Florin et al. (2012) who used partial directed coherence analysis to conclude that 4–8 Hz rhythms are propagated from the periphery to the center.

Alternatively, the spike-doublets of PD tremor could be due to “successive facilitatory stimuli” as suggested by Das Gupta (1963) for the generation of force tremor in PD. If this were the case, the herein observed doublets/triplets would reflect rhythmical, beta range, MN inputs from a tremor generator in the brain. Consistent with this, the average short ISI is in the middle of the values expected of the period of beta range oscillations. This is not the first time that beta-rhythms have been implicated in the generation of tremor (e.g., Levy et al., 2002; Brown, 2003). By analogy to the high frequency oscillations of phrenic MNs in response to medullary descending inputs (Christakos et al., 1988) and in line with a previous hypothesis from this laboratory (Christakos et al., 2009), the periodic beta-range input would ride on top of a slower rhythm such as that underlying physiological tremor. The combination of the two input rhythms would push the envelope of MN membrane potential modulation toward smaller frequencies (4–8 Hz; see next subsection, below) and give rise to rhythmical local peaks, ca. 40 ms apart, in each cycle. The membrane potential of the MN would thus repeatedly surpass (2–4 times/cycle) the threshold for initiating action potentials. Coherent beta-rhythms have been observed in cerebello-thalamo-cortical loops (Timmermann et al., 2003) showing coherence to sEMG records (Marsden et al., 2000). A recent hypothesis implicates this loop in the genesis of PD tremor (Helmich et al., 2012) and ascribes to the basal ganglia a role as a parkinsonian tremor “switch,” intermittently triggering activity in the cerebello-thalamo-cortical loop and thus the transition to epoch-II intervals. Similar beta-range oscillations have been found in low frequency potentials recorded from the subthalamic nucleus, and were patient specific enough to merit the term “signature rhythm” (Bronte-Stewart et al., 2009). Finally, deep-brain stimulation of the ventral intermediate thalamic nucleus (Fukuda et al., 2004), leading to reduction of cortical and cerebellar activity, causes drastic decrease in the amplitude of tremor and increases its frequency from abnormal (4–8 Hz) to normal (6–10 Hz) values, i.e., changes similar to those that take place during transitions from epoch-II to epoch-I intervals. Similarly, deep-brain stimulation of the subthalamic nucleus causes tremor attenuation (Bronte-Stewart et al., 2009) through cortical-beta suppression (Menon et al., 2013).

### Possible Role of the Spinal Stretch Reflex Loop in Parkinsonian Tremor

Earlier proposals implicating the involvement of the stretch reflex loop in the generation of tremor in PD patients were considered in the Section “Introduction.” This loop has been also implicated in the generation of the physiological tremor which depends on spindle sensitivity and associated MU synchrony (Lippold et al., 1957; Lippold, 1970; Hagbarth and Young, 1979). Strong supportive evidence for the crucial role of spindle feedback in the generation of physiological tremor was more recently provided through the ischemic Ia afferent block suppressing both synchrony and tremor (Christakos et al., 2006; Erimaki and Christakos, 2008).

**FIGURE 9:**
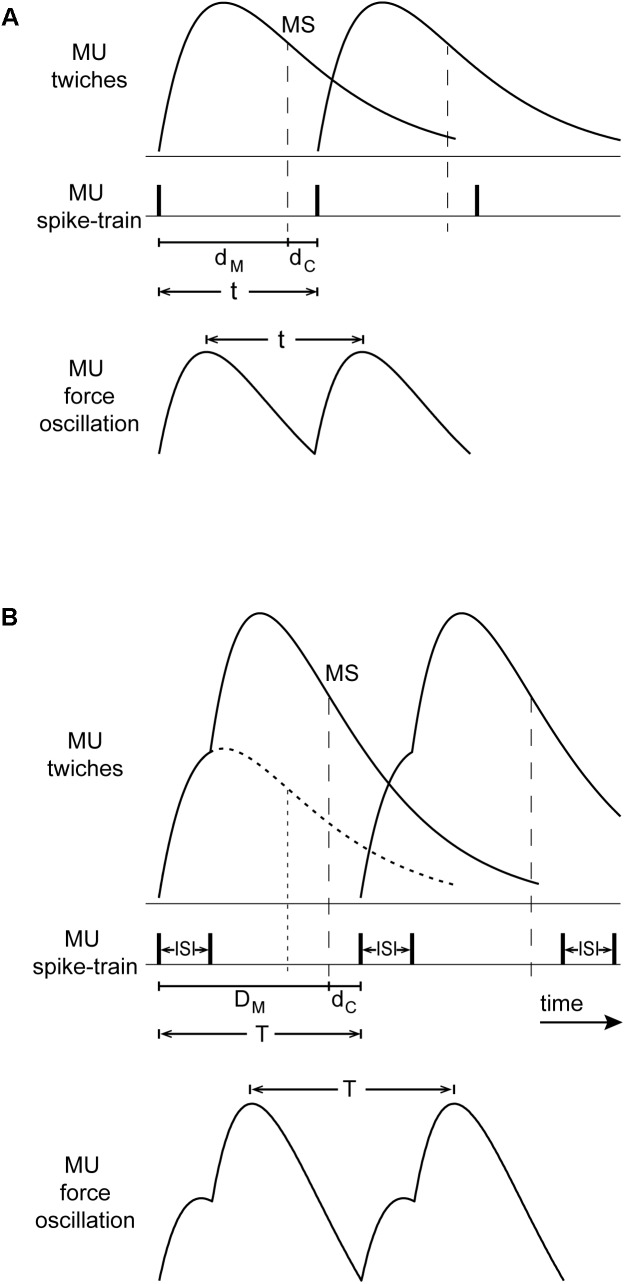
Conceptual model of the interaction between the stretch reflex loop and descending high-frequency oscillations that could account for our observations. **(A)** Top: MU-twitches resulting from rhythmical discharges and associated muscle spindle discharge during the decaying phase of the MU-twitch (MS, vertical dashed line, i.e., occurring at peak lengthening velocity); contraction delay *d*_M_, from the onset of this MU-twitch to the moment of spindle discharge, and the conduction delay *d*_C_ (afferent and efferent) from MS to the onset of the subsequent MU-twitch. Bottom: The period of the resulting MU force oscillation *t*, is equal to the sum of *d*_M_ and *d*_C_. **(B)** Top: large and broadened force-pulses resulting from the summation of two MU-twitches (solid line), caused by spike-doublets characterized by a 40 ms interspike interval (ISI); for comparison, the profile of a single MU-twitch is depicted as dotted line; the contraction delay *D*_M_ is thereby prolonged and its decay phase slope steeper as compared with that of the single twitch. Bottom: the period of the resulting MU force oscillation *T*, is in this case prolonged and its amplitude increased.

Oscillations due to the operation of the stretch reflex loop occur at a frequency corresponding to the *overall delay* around the loop which normally consists of (i) a roughly 100-ms *muscle contraction delay* from the onset of lumped MU-twitches to the onset of grouped muscle spindle discharges during the decay of the muscle-twitch, i.e., around the time of peak lengthening velocity (Lippold et al., 1957), and (ii) a roughly 30-ms afferent and efferent *conduction delay*. The resulting 130 ms overall delay corresponds to a tremor frequency of 7.7 Hz (Oppenheim et al., 1983), which lies roughly in the middle of the 6–10 Hz range of physiological tremor frequencies (Christakos et al., 2006) as well as, of non-overt tremor frequencies during epoch-I intervals in PD patients. **Figure 9A** illustrates a schematic representation of rhythmic spikes, force-twitches and force oscillation of a hypothetical MU during epoch-I intervals.

During epoch-II intervals, it is MUs with relatively high *f*_U_ that are likely to contribute to the tremor. They emit spikes about 40 ms apart which are grouped into doublets and triplets. The summation of their twitches results into ‘broadened’ force-pulses (**Figure 9B**). Because of the increase of the contraction delay, the overall delay increases proportionately (**Figure 9B**). As a consequence, the frequency of the primary component of tremor would drop to the 4–8 Hz range that characterizes PD. This line of argument is supported by the positive correlation between the mean short ISI and the difference *T*_P_–*T*_S_, where *T*_P_ is the period of the primary component of the tremor and *T*_S_ that of the secondary. Likewise, the presence of broader MU-twitches in leg muscles (Belanger and McComas, 1985) relative to those in arm muscles could be responsible for the lower frequency of parkinsonian leg tremor, compared with that of arm tremor (O’Suilleabhain and Matsumoto, 1998). Moreover, summation of two (doublet) or more (triplet, quadruplet) twitches of the same MU leads to contraction decays with steeper slope (**Figure 9B**; Figure 3 of Elek et al., 1991; Figure 2 of Thomas et al., 1999). In turn, muscle spindles respond more vigorously, leading to enhanced MU synchrony and additional tremor enhancement. Thus, the occurrence of spike doublets/triplets leads to a reduction of tremor frequency and increase tremor amplitude.

## Conclusion

Parkinsonian tremor exhibits an abnormal, low frequency component (4–8 Hz), which is abnormally large due to the presence of enhanced MU synchrony and spike doublets/triplets with beta-range (30–50 ms) mean ISIs and is randomly interspersed within segments of higher frequency (6–10 Hz) physiological tremor. In each patient, the frequencies of the two components differ by about 1.5 Hz, but also the synchrony enhancement and the mean ISIs, are the same for all tremor types; moreover, these fixed, patient-specific mean ISIs seem to link the two generators. These data, are consistent with a neural origin of PD tremor and severely constrain models of its generation, whether cerebral or spinal-peripheral. The spinal stretch reflex loop, acting as a two-state oscillator influenced by intermittent, descending high-frequency (beta-range) oscillations, could explain our data as well as previous observations on tremor-related cerebral rhythms. Finally, our analysis of MU activity links tremor appearance to underlying neural generators and could facilitate tremor studies in other movement disorders.

## Author Note

Constantinos N. Christakos deceased on June 15th 2015. This article was seen and approved by him in early June 2015.

## Author Contributions

OA, CC, and DA participated in the various aspects of the work and substantially contributed to the conception and design. DA and OMA performed the data collection. OA and CC analyzed and interpreted the data. All the authors wrote and critically reviewed the manuscript and approved the final version and agreed to be accountable for all aspects of the work in ensuring that questions related to the accuracy or integrity of any part of the work are appropriately investigated and resolved.

## Conflict of Interest Statement

The authors declare that the research was conducted in the absence of any commercial or financial relationships that could be construed as a potential conflict of interest.

## Abbreviations

*f*_T_: frequency of overt tremor
*f*_U_: mean firing rate of motor unit
ISI: interspike interval
MN: motoneuron
MU: motor unit
PD: Parkinson’s disease
sEMG: surface EMG
